# The 5-HT_6_ Receptors in the Ventrolateral Orbital Cortex Attenuate Allodynia in a Rodent Model of Neuropathic Pain

**DOI:** 10.3389/fnins.2020.00884

**Published:** 2020-08-18

**Authors:** Yuxiang Zhang, Jingsi Yang, Xixi Yang, Yanan Wu, Junlin Liu, Yangdong Wang, Fuquan Huo, Chunxia Yan

**Affiliations:** ^1^College of Forensic Medicine, Xi’an Jiaotong University Health Science Center, Xi’an, China; ^2^The Key Laboratory of Environment and Genes Related to Diseases, Ministry of Education, Xi’an Jiaotong University, Xi’an, China; ^3^Department of Physiology and Pathophysiology, School of Basic Medical Sciences, Xi’an Jiaotong University Health Science Center, Xi’an, China

**Keywords:** 5-HT_6_ receptors, neuropathic pain, mechanical allodynia, AC, PKA, anti-nociception, ventrolateral orbital cortex

## Abstract

Mechanical allodynia, characterized by a painful sensation induced by innocuous stimuli, is thought to be caused by disruption in pain-related regions. Identification and reversal of this pathologic neuroadaptation are therefore beneficial for clinical treatment. Previous evidence suggests that 5-HT_6_ receptors in the ventrolateral orbital cortex (VLO) are involved in neuropathic pain, but their function is poorly understood. The aim of the present study is to unveil the role of 5-HT_6_ receptors in the VLO and the underlying mechanisms in pain modulation. Here, by using the spared nerve injury (SNI) pain model, first, we report that 5-HT_6_ receptor protein decreased in the contralateral VLO compared with the ipsilateral VLO in rats with allodynia. Second, microinjection of the selective 5-HT_6_ receptor agonists EMD-386088 and WAY-208466 into the contralateral VLO consistently and significantly depressed allodynia. Third, microinjection of the selective antagonist SB-258585 blocked the agonist-induced anti-allodynic effect, while the antagonist applied alone to the VLO had no effect. Furthermore, the anti-nociceptive effect of EMD-386088 on neuropathic pain was prevented by the adenylate cyclase (AC) inhibitor SQ-22536, and protein kinase A (PKA) inhibitor H89, suggesting that AC/PKA signaling might underlie the antinociception of agonists. Finally, the 5-HT_6_ receptors were found to be colocalized with a glutamate transporter (EAAC1) by immunofluorescent staining, and the glutamate receptor antagonist kynurenic acid was found to completely block antinociception. These findings indicated that the antinociceptive effect of 5-HT_6_ receptor agonists might occur via interaction with the glutamatergic system. Altogether, the agonists activated 5-HT_6_ receptors present in the glutamatergic neurons in the VLO to facilitate the AC/PKA cascade, which subsequently might evoke glutamate release, thus depressing allodynia. These findings suggest a potential therapeutic role of 5-HT_6_ receptor agonists in treating neuropathic pain.

## Introduction

Neuropathic pain is a maladaptive pain condition that has become a public health challenge (Gomez et al., 2020). This condition can develop as a result of injury or nerve illness. Mechanical allodynia, a misperception of light touch as painful (Lee et al., 2019), occurs when normal processing in the central nervous system is compromised. The ventrolateral orbital cortex (VLO), one of the most crucial parts of the prefrontal cortex, together with the nucleus submedius (Sm) and the midbrain periaqueductal gray (PAG), constitutes a pain modulatory pathway. Under some pathological conditions, for instance, acute persistent inflammatory pain and neuropathic pain, activation of the VLO leads to activation of the PAG–brainstem descending inhibitory system and consequently depresses nociceptive inputs into the spinal cord and trigeminal nucleus, thus resulting in anti-nociception (Tang et al., 2009).

Morphological results have revealed that the VLO receives serotonin (5-hydroxytryptamine, 5-HT) fibers from the dorsal raphe nucleus (DR) (Li et al., 1993; Matsuzaki et al., 1993; Huo et al., 2009). Growing evidence supports a fundamental role of 5-HT in pain processing and regulation via its interaction with different 5-HT receptors (Bardin et al., 2001; Cortes-Altamirano et al., 2018). Among the recently discovered 5-HT receptors, the 5-HT_6_ subtype is of special interest. Mounting evidence suggests that both 5-HT_6_ receptor agonists and antagonists display pro-cognitive properties (Hatat et al., 2019; Latacz et al., 2019; Rychtyk et al., 2019; Shahidi et al., 2019), such as improving memory impairment. Similarly, several antipsychotic and antidepressant drugs have high affinity for 5-HT_6_ receptors (Kim et al., 2019), and 5-HT_6_ receptor agonists and antagonists both induce anxiolytic- and antidepressant-like effects (Yun et al., 2015; Suarez-Santiago et al., 2017; Chaumont-Dubel et al., 2019). Such paradoxical but intriguing findings have ignited tremendous exploration of the underlying molecular signaling. Apart from coupling to a stimulatory Gα protein, which increases cyclic adenosine monophosphate (cAMP) formation and then activates cAMP-dependent protein kinase A (PKA), 5-HT_6_ receptors also interact with diverse partners, and specific downstream pathways might underlie different effects of agonists or antagonists at different physiological and pathological settings (Yun et al., 2007, 2010; Kim et al., 2014; Kim et al., 2019). A pioneering finding about 5-HT_6_ receptors on neuropathic pain suggests that exogenous 5-HT entering the VLO can potentially depress mechanical allodynia and that this effect can be blocked in part by 5-HT_6_ receptor antagonists (Xu et al., 2013). Although these findings imply that 5-HT_6_ receptors in the VLO might be involved in pain modulation, their roles and underlying mechanisms are not yet fully elucidated.

Therefore, the present study was conducted (i) to examine the role of 5-HT_6_ receptors in the VLO in neuropathic pain model and (ii) to examine the mechanistic underpinnings of 5-HT_6_ receptors affecting nociceptive behaviors. Using this framework, we speculate on whether 5-HT_6_ receptors represent a platform for the development of therapeutic interventions in neuropathic pain.

## Materials and Methods

### Animals

Male Sprague-Dawley rats (250–300 g) were used for all experiments and were provided by the Medical Experimental Animal Center of Xi’an Jiaotong University, Shaanxi Province, China. The animals were group housed under a 12-h/12-h light/dark cycle with access to food and water *ad libitum*. All animals were acclimatized for 3 days before any experimentation. The experimental protocols and animal procedures were approved by the Institutional Animal Care Committee of Xi’an Jiaotong University and were carried out in strict accordance with the ethical guidelines of the International Association for the Study of Pain (Zimmermann, 1983) for the care and use of laboratory animals. Best effort was made to minimize the number of animals used as well as their suffering.

### Spared Sciatic Nerve Injury

**FIGURE 1:**
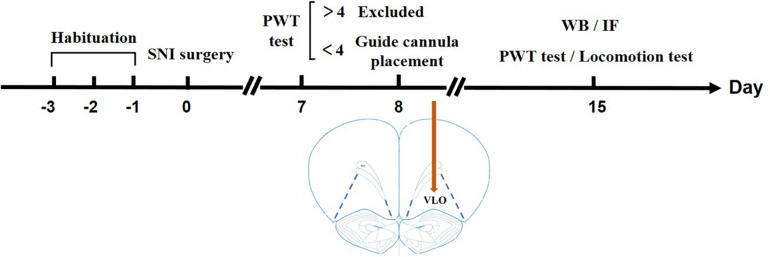
Timeline illustrating a summary of the experimental design. After habituation for 3 days, SNI surgery was performed on Day 0. On Day 7, a PWT test was performed to measure if the rats developed allodynia. If the PWT > 4.0 g, the rats were excluded from later experiments; if the PWT < 4.0 g, a guide cannula was placed in the VLO. After the surgery, the rats were in their homecage for 7 days to recover until Day 14. On Day 15, a second PWT or locomotion test was performed to measure the effect of drugs. For Western blotting or immunofluorescent staining, the rats were sacrificed on Day 15 without cannula placement.

The spared nerve injury (SNI) model was implemented as previously described (Decosterd and Woolf, 2000; Arsenault and Sawynok, 2009; Shiers et al., 2020). Briefly, the rats were intraperitoneally (i.p.) anesthetized with sodium pentobarbital (50 mg/kg, Sinopharm Chemical Reagent Co., Ltd., Shanghai, China) and then an incision was made along the left back of the thigh to expose the sciatic nerve and peripheral branches (common peroneal, tibial, and sural nerves). Next, tight ligation of the common peroneal and tibial nerves was performed by using 4.0 silk suture. The ligated nerves were transected distally and a 2 mm section was removed to prevent nerve regeneration, while the sural nerve was left intact, and then the wound was closed. During sham (control) surgery, the three branches of the sciatic nerve were exposed, but no ligation or transection was performed. Following surgery, the rats were carefully nursed to recover for 7 days before implantation of intracerebral guide cannulas. The overall experimental schedule is shown in Figure 1.

### Intracerebral Guide Cannula Placement

The rats were anesthetized through i.p. injection of sodium pentobarbital (50 mg/kg). Then the head was immobilized in a stereotaxic frame. A small craniotomy was performed just above the VLO, contralateral to the affected paw, since a majority of the nociceptive signals are transmitted to the contralateral VLO. The coordinates for the unilateral VLO microinjection were as follows: AP + 4.2 mm, ML + 2.0 mm, DV – 4.6 mm (Paxinos, 1997). A stainless steel guide cannula was stereotaxically inserted (Huo et al., 2008). Once the rats recovered from anesthesia, they were i.p. injected with sodium penicillin (0.2 million U/day for 3 days) to prevent wound and intracerebral infection. The rats were then carefully nursed and housed in clean cages for 7 days before the behavioral test.

### Mechanical Paw Withdrawal Threshold (PWT) Measurement

The paw withdrawal threshold (PWT) was quantified in response to mechanical stimulation (von Frey filaments) using the up-down method (Dixon, 1980; Chaplan et al., 1994). The animals were gently placed in a transparent plastic box with a metal wire mesh floor, which allowed full access to the paws from below. Ten von Frey filaments (Stoelting Company, Wood Dale, IL, United States), with approximately equal logarithmic incremental (0.17) bending force levels, were chosen (von Frey numbers: 3.61, 3.84, 4.08, 4.17, 4.31, 4.56, 4.74, 4.93, 5.07, and 5.18, equivalent to: 0.4, 0.6, 1.0, 1.4, 2.0, 4.0, 6.0, 8.0, 10, and 15.0 g, respectively). Starting with filament 4.31 (2.0 g), which was the middle filament in the series, von Frey filaments with different intensities were repeatedly applied over a 2-s time interval from below and perpendicular to the fourth and fifth toes of the hind paw with sufficient force to cause slight bending against the paw for 6–8 s. If the response to filament stimulation was positive, the next lower force was delivered. If there was no withdrawal response (negative), the next higher force was utilized. Positive and negative responses were recorded and converted to a 50% threshold using a formula provided by Dixon (1980) and Chaplan et al. (1994). PWT measurements were performed in 10-min intervals over a 60-min observation period. Mechanical allodynia was considered to be successful if the PWT was reduced to <4.0 g. The experimenters performing behavioral experiments were blinded to drug treatments during the behavioral assessment.

### Intracerebral Microinjection of Drugs

Rats were lightly anesthetized with enflurane (Baxter Caribe, Guayama, PR, United States), a very short-acting inhalation anesthetic (approximately 1–2 min), and a 1-μl microsyringe with the tip extending 2 mm beyond the end of the guide cannula was inserted into the VLO through the guide cannula. For double-administered agents, the interval between the two injection was 10 min. Over a 60-s period, 0.5 μl of drug dissolved in saline or 10% dimethyl sulfoxide (DMSO) was slowly infused at constant speed, and after the rats recovered from the enflurane anesthetized, effects on allodynia were observed over a 60-min period following drug infusion. Values from each treated group were obtained from different experiments (Zhu et al., 2013). The following drugs were used in this study: selective 5-HT_6_ receptor agonists EMD-386088 (Pratt et al., 2012) and WAY-208466 (Liu et al., 2015), selective 5-HT_6_ receptor antagonist SB-258585 (Liu et al., 2015), non-selective glutamate receptor (NMDA and AMPA/kainate receptors) antagonist kynurenic acid (KA) (Braccialli et al., 2008), adenylate cyclase (AC) inhibitor SQ-22536 (SQ) (Li et al., 2013), and PKA inhibitor H89 (Khorshidahmad et al., 2012; Guo et al., 2017). WAY-208466 and SB-258585 were purchased from RBI/Sigma (St. Louis, MO, United States), while EMD-386088, SQ and H89 were obtained from Tocris Cookson (Bristol, United Kingdom). All the drugs were dissolved in saline or 10% DMSO. The agonist was injected into the VLO, contralateral to the affected hind paw, and the antagonist was administered 10 min prior to the agonist. The effective drug doses were chosen according to previous studies (Braccialli et al., 2008; Khorshidahmad et al., 2012; Pratt et al., 2012; Li et al., 2013; Liu et al., 2015) and our preliminary experiments. Equal volumes of 10% DMSO were injected into the VLO and served as the vehicle.

### Open-Field Test

To explore whether microinjection of 5-HT_6_ receptor agonists into the VLO affects the locomotor activity of animals, an open-field test was conducted after administration of the selective 5-HT_6_ receptor agonist EMD-386088 or the vehicle into the VLO of the SNI rats. Video-tracking software (SMART, Panlab SL, Barcelona, Spain) was used to test individual rats. Testing boxes (60 × 60 × 40 cm^3^, length × width × height) were set in an isolated dark room, and two standard laboratory lamps were fixed above the boxes for illumination. The room was kept quiet and dimly lit during the whole experiment. After the rats were gently placed in the boxes, their locomotor activities were recorded by a digital camera (SONY) and analyzed offline by the horizontal trajectories reflecting the animals’ locomotor activity. The videos were recorded using digital video cameras mounted above the testing boxes.

### Western Blotting

Rats went through procedures in Figure 1 without cannula placement, microinjection or locomotion test. Western blotting was performed as previously described (Zhang et al., 2018). Briefly, brain tissue containing the VLO was rapidly collected after animal anesthesia and decapitation. The tissue was dissected and homogenized in ice-cold lysis buffer. The homogenate was incubated on ice for 30 min and then centrifuged at 10,000 *g* for 15 min at 4°C. The supernatant was collected and stored at −80°C until use. Sample protein levels were measured using a bicinchoninic acid (BCA) protein assay. Loading proteins were separated on acrylamide gels and then transferred to pore size 0.45 μm polyvinylidene difluoride (PVDF) membranes (Millipore, Billerica, MA, United States). After blocking with 5% non-fat milk, the membranes were probed for anti-rabbit 5-HT_6_ receptor (Abcam, ab103016, 1:1,500, Cambridge, MA, United States), and anti-mouse actin (Sigma, A5316, 1:100,000, St Louis, MO, United States). Densitometry of each protein was evaluated by Image-J (NIH). Samples from each animal were run at least four times to minimize inter-blot variance. Raw values were normalized to actin.

### Histology, Immunofluorescence and Image Analysis

After the behavioral experiment, the animals were deeply anesthetized with chloral hydrate (400 mg/kg) and the microinjection sites were marked with Pontamine Sky Blue dye (0.5 μl, 2% in 0.5 M sodium acetate solution). Rats were transcardially perfused with 0.01 M PBS (pH 7.4), followed by 4% paraformaldehyde solution (PFA, pH 7.4). The brains were then removed and fixed in 4% PFA for 24 h, then placed in 30% sucrose solution for 3 days at room temperature. Subsequently, the brains were embedded in optimum cutting temperature medium (Tissue Tek OCT; Sakura) and cut into 40 μm thick sections using a Leica CM3050 S cryostat. Then the slices were mounted on gelatin-covered slides and stained with cresyl violet. The injection sites were histologically identified to be within the VLO or not. Only the data with explicit corresponding histology representing presence within the VLO were used for final data analysis and 15 rats were excluded from data analysis. To keep consistent, we dissect it from bregma +4.2 to + 3.24 (Wei et al., 2016). A schematic representation and photomicrograph of cresyl violet staining in the VLO are shown in Supplementary Figures S1, S2.

For immunofluorescence, rats went through procedures in Figure 1 without cannula placement, microinjection or locomotion test. Similarly, rats were perfused with 0.01 M PBS and 4% PFA after deep anesthetization. The brains were then removed, fixed overnight, transferred to 30% sucrose, and stored at 4°C. Coronal sections (30 μm) containing the VLO were cut in a cryostat for free floating staining. After washing in PBS, sections were incubated with primary antibody anti-rabbit 5-HT_6_ receptor (Bioss, bs-12058R, 1:1,000, Beijing, China) and anti-goat neuronal glutamate transporter (EMD Millipore, AB1520, 1:8,000, Temecula, CA, United States) for 48 h at 4°C. Fluorescent secondary antibodies (Invitrogen/Thermo Fisher Scientific), goat anti-rabbit Alexa 488 and donkey anti-goat CY3 at dilutions of 1:500 and 1:600, respectively, were incubated for 2 h at room temperature. Coverslips were mounted using mounting media with DAPI (Abcam, ab104139). Sections were stained at the same time and images of the VLO were taken with the same exposure time. By using Image-J, the intensity threshold was applied to each image to include the positive labeling while minimizing the inclusion of non-specific, background staining in the sections. The experimenters were blind to the experimental conditions during imaging and analysis.

### Data Analysis

All values were expressed as mean ± SEM. One-way ANOVA or two-way ANOVA analyses were performed using IBM SPSS statistics 19.0 (SPSS Inc., Chicago, IL, United States). The areas under the time-course curve (AUC) of the PWT during the 60-min observation period were counted. For results with significant interaction effects in two-way ANOVA, a simple effect test was conducted for further analysis. For results without significant interaction effects, Bonferroni’s *post hoc* test or Student’s *t*-test was further conducted as needed. To determine differences between two groups, Student’s *t*-test was performed. *P* < 0.05 was considered to be statistically significant. All values were expressed as mean ± SEM.

## Results

**FIGURE 2:**
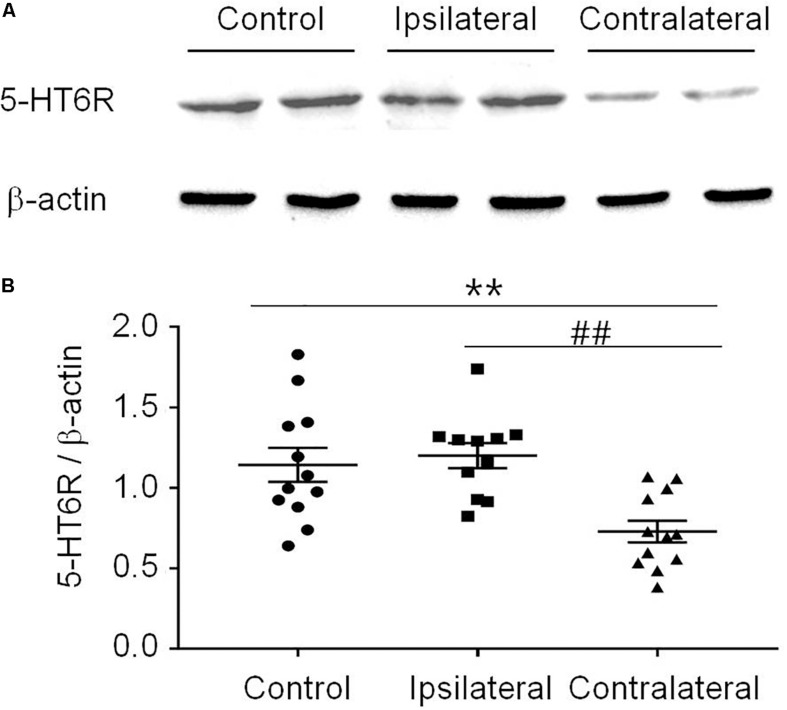
The expression of 5-HT_6_ receptors protein level was specifically decreased in the contralateral VLO after SNI. **(A)** Representative Western blotting and **(B)** summary scatter plots showing the 5-HT_6_ receptors of the VLO in control (sham) and SNI rats. **p < 0.01 and ^##^*p* < 0.01 vs corresponding control and contralateral group, respectively. *n* = 4 rats/group. 5-HT6R, 5-HT_6_ receptors.

**FIGURE 3:**
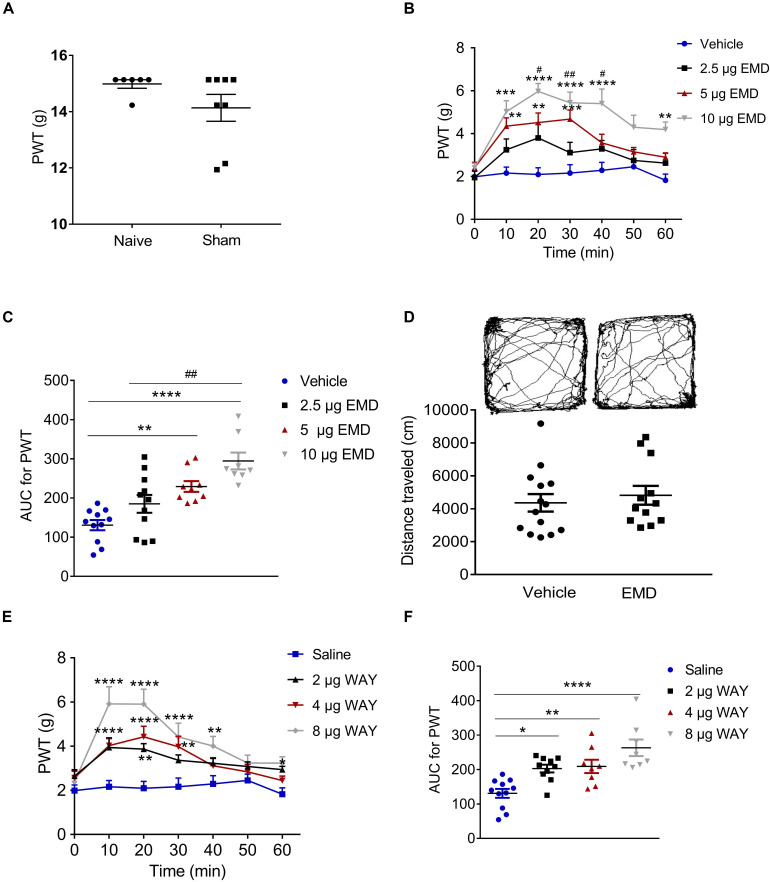
Microinjection of the selective 5-HT_6_ receptor agonists into the contralateral VLO reduced SNI-induced allodynia. **(A)** Scatter plots showing no difference of PWT between naïve and sham animals. **(B,E)** Time-course curves showing the inhibitory effect of different doses of EMD-386088 and WAY-208466 on allodynia induced by SNI. **(C,F)** Scatter plots showing the inhibitory effect of different doses of EMD-386088 and WAY-208466 on allodynia during the 60-min observation period. **(D)** The locomotor activity in an open-field test was not affected after microinjection of 10 μg EMD-386088 into the VLO. *p < 0.05, **p < 0.01, ***p < 0.001, and ****p < 0.0001 compared with the vehicle group; ^#^*p* < 0.05, ^##^*p* < 0.01, and ^###^*p* < 0.001, compared with the 2.5 μg EMD group. *n* = 6–15 rats/group.

*T*-test (*t*_12_ = 1.483, *p* = 0.1640) showed that the PWT in the sham group (14.14 ± 0.4773) was not different from that in the naïve group (14.98 ± 0.1509). Therefore, the sham was used as the control in the following experiments (Figure 3A). Similar to previous reports (Decosterd and Woolf, 2000; Dang et al., 2010), the PWT decreased below 4.0 g 2 weeks after rat SNI induction, compared to 15.0 g in rats with sham SNI induction. To examine the possible role of 5-HT_6_ receptors in neuropathic pain, we first measured the expression level of these receptors in the VLO in control and SNI rats. For the SNI rats, we further separated the contralateral VLO from the ipsilateral VLO. One-way ANOVA analysis indicated a significant effect [*F*_(__2_, _32__)_ = 9.156, *p* = 0.0007], and *post hoc* analyses showed a significantly lower protein level of 5-HT_6_ receptors in the VLO contralateral to the affected paw (0.729 ± 0.067), compared to those of the ipsilateral VLO (1.201 ± 0.078, *p* = 0.0084) and control (1.143 ± 0.105, *p* = 0.0090) groups (Figure 2). These results suggested that SNI specifically decreased the expression of 5-HT_6_ receptors in the contralateral VLO but not in the ipsilateral VLO.

### Anti-nociception of 5-HT_6_ Receptor Agonist EMD-386088 in the VLO of SNI-Induced Allodynia

Considering that SNI induced a specific inhibitory effect on 5-HT_6_ receptor expression in the contralateral VLO, we first microinjected selective 5-HT_6_ receptor agonist EMD-386088 (2.5, 5, and 10 μg, respectively) into the contralateral VLO. We found that EMD-386088 (5 and 10 μg) reduced SNI-induced allodynia as the PWT was increased (Figures 3B,C). As shown in Figure 3B, time course curves of PWT for the vehicle and different doses of the EMD-386088 treated groups were different between treatments [*F*_(__3_, _210__)_ = 13.40, *p* < 0.0001], across times [*F*_(__6_, _210__)_ = 16.96, *p* < 0.0001] and for treatment × time interaction [*F*_(__18_, _210__)_ = 2.698, *p* < 0.0001]. The detailed comparisons at individual time points between various groups are also shown in Figure 3B (1.987 ± 0.257, 2.163 ± 0.282, 2.096 ± 0.310, 2.161 ± 0.393, 2.291 ± 0.373, 2.453 ± 0.292, and 1.824 ± 0.291 for vehicle group; 1.952 ± 0.303, 3.246 ± 0.504, 3.800 ± 0.620, 3.114 ± 0.480, 3.293 ± 0.391, 2.747 ± 0.511, and 2.626 ± 0.460 for 2.5 μg EMD group; 2.413 ± 0.246, 4.351 ± 0.386, 4.518 ± 0.446, 4.678 ± 0.425, 3.567 ± 0.402, 3.151 ± 0.204, and 2.901 ± 0.207 for 5 μg EMD; 2.401 ± 0.215, 5.024 ± 0.510, 5.969 ± 0.371, 5.440 ± 0.500, 5.401 ± 0.680, 4.299 ± 0.566, and 4.191 ± 0.351 for 10 μg EMD). Further analyses of the AUC of the PWT during the 60-min observation period showed that the inhibitory effects induced by 5 and 10 μg on allodynia were significantly larger than in the vehicle groups (*p* < 0.01 and *p* < 0.0001, respectively). In addition, the effects of 10 μg EMD-386088 were significantly larger than the 2.5 μg group (*p* < 0.01). No significant difference was observed between the 5 and 10 μg EMD-386088 groups, as shown in Figure 3C (130.758 ± 2.930 for vehicle group; 184.976 ± 22.815 for 2.5 μg EMD group; 229.310 ± 13.815 for 5 μg EMD; 294.375 ± 21.543 for 10 μg EMD). In the sham rats, we did not observe any effect of 5-HT_6_ receptor agonist EMD-386088 in the VLO on PWT (Supplementary Figure S3).

### No Effect of 5-HT_6_ Receptor Agonist EMD-386088 in the VLO on Basal Locomotion

Next, we examined the potential effects of EMD-386088 on basal locomotor activity. In the open field test, the locomotor activity of the SNI rats after microinjection of EMD-386088 (10 μg, 4819.301 ± 570.436) or the vehicle (4358.614 ± 533.707) into the contralateral VLO was not significantly different between the two groups during the 60-min observation period (*p* > 0.05, Figure 3D). These results corroborated our findings shown in Figure 3C, further demonstrating that EMD-386088 was able to attenuate SNI-induced allodynia without influencing exploration in the test apparatus.

### Anti-nociception of 5-HT_6_ Receptor Agonist WAY-208466 in the VLO of SNI-Induced Allodynia

However, a previous study also reported a weaker effect of EMD-386088 on 5-HT_3_ receptors and dopamine reuptake (Mattsson et al., 2005; Jastrzębska-Więsek et al., 2016). To further confirm the effect of activation of 5-HT_6_ receptors, we microinjected another selective 5-HT_6_ receptor agonist, WAY-208466, into the contralateral VLO. Similarly, WAY-208466 (2, 4 and 8 μg) reduced SNI-induced allodynia as the PWT was increased (Figures 3E,F). As shown in Figure 3E, the time course curves of the PWT for saline and different doses of the WAY-208466-treated groups were different between treatments [*F*_(__3_, _231__)_ = 37.90, *p* < 0.0001], across times [*F*_(__6_, _231__)_ = 12.71, *p* < 0.0001] and for treatment × time interaction [*F*_(__18_, _231__)_ = 2.539, *p* = 0.0008]. Detailed comparisons at individual time points between the various groups are shown in Figure 3E (1.987 ± 0.257, 2.163 ± 0.282, 2.096 ± 0.310, 2.161 ± 0.393, 2.291 ± 0.373, 2.453 ± 0.292, and 1.824 ± 0.291 for saline group; 2.648 ± 0.287, 3.940 ± 0.438, 3.866 ± 0.249, 3.363 ± 0.241, 3.225 ± 0.221, 3.082 ± 0.210, and 2.941 ± 0.1462 for 2 μg WAY group; 2.569 ± 0.300, 4.026 ± 0.323, 4.424 ± 0.478, 3.976 ± 0.471, 3.115 ± 0.365, 2.836 ± 0.336, and 2.445 ± 0.206 for 4 μg WAY group; 2.388 ± 0.226, 5.915 ± 0.779, 5.903 ± 0.677, 4.421 ± 0.627, 4.011 ± 0.436, 3.235 ± 0.356, and 3.231 ± 0.287 for 8 μg WAY group). Further analyses of the AUC of the PWT during the 60-min observation period showed that the inhibitory effects induced by 2, 4, and 8 μg on allodynia were significantly larger than in the vehicle group (*p* < 0.05, *p* < 0.01, and *p* < 0.0001, respectively). No significant difference was observed between these groups, as shown in Figure 3F (130.758 ± 12.930 for saline group; 202.823 ± 11.147 for 2 μg WAY group; 208.949 ± 19.442 for 4 μg WAY group; 262.997 ± 24.083 for 8 μg WAY group). These findings demonstrated that the inhibitory effect of 5-HT_6_ receptor agonists on allodynia was due to the activation of 5-HT_6_ receptors.

### Antagonizing Effect of SB-258585 on EMD and WAY-Induced Anti-allodynia

**FIGURE 4:**
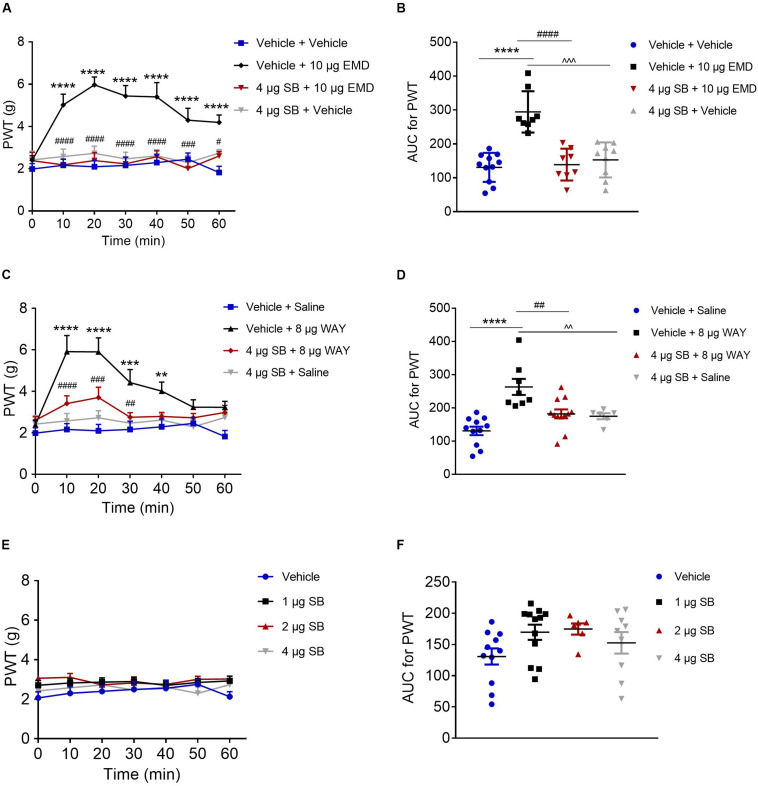
Effects of the selective 5-HT_6_ receptor antagonist SB-258585 on EMD- and WAY-induced anti-allodynia. **(A,C)** Time course curves showing the antagonizing effect of SB-258585 on EMD- and WAY-induced inhibition of allodynia. **(B,D)** Scatter plots showing the antagonizing effect of SB-258585 on EMD- and WAY-induced inhibition of allodynia during the 60-min observation period. **(E)** Time-course curves showing no effect of different doses of SB-258585 on allodynia induced by SNI. **(F)** Scatter plots showing no effect of different doses of SB-258585 on allodynia during the 60-min observation period. **p < 0.01, ***p < 0.001, and ****p < 0.0001 compared with the vehicle group; ^#^*p* < 0.05, ^##^*p* < 0.01, ^###^*p* < 0.001, and ^####^*p* < 0.0001 compared with the 10 μg EMD and 8 μg WAY group. *n* = 6–12 rats/group.

Microinjection of the selective 5-HT_6_ receptor antagonist SB-258585 (4 μg) into the VLO, 10 min prior to EMD-386088 (10 μg) injection, completely antagonized the EMD-evoked inhibition of allodynia as the PWT was reduced to the control level, as shown in Figures 4A,B. The time course curves of the PWT in the vehicle + vehicle, vehicle + EMD, SB + EMD, and SB + vehicle groups were different between treatments [*F*_(__3_, _224__)_ = 69.73, *p* < 0.0001], across times [*F*_(__6_, _224__)_ = 3.388, *p* = 0.0032] and for treatment × time interaction [*F*_(__18_, _224__)_ = 2.546, *p* = 0.0008]. Detailed comparisons at individual time points between various groups were shown in Figure 4A (1.987 ± 0.257, 2.163 ± 0.282, 2.096 ± 0.310, 2.161 ± 0.393, 2.291 ± 0.373, 2.453 ± 0.292, and 1.824 ± 0.291 for vehicle + vehicle group; 2.401 ± 0.215, 5.024 ± 0.510, 5.969 ± 0.371, 5.440 ± 0.500, 5.401 ± 0.680, 4.299 ± 0.566, and 4.191 ± 0.351 for vehicle + EMD group; 2.384 ± 0.395, 2.183 ± 0.280, 2.386 ± 0.372, 2.233 ± 0.313, 2.569 ± 0.279, 2.009 ± 0.406, and 2.605 ± 0.205 for SB + EMD group; 2.421 ± 0.413, 2.577 ± 0.356, 2.726 ± 0.342, 2.468 ± 0.315, 2.612 ± 0.295, 2.292 ± 0.341, and 2.739 ± 0.168 for SB + vehicle). Further analyses indicated that the AUC in the SB + EMD group was significantly smaller than in the vehicle + EMD group (*p* < 0.0001), and no significant difference was found between the SB + EMD and vehicle + vehicle groups, as shown in Figure 4B (130.758 ± 12.930 for vehicle + vehicle group; 294.375 ± 21.543 for vehicle + EMD group; 138.823 ± 16.617 for SB + EMD group; 152.618 ± 17.212 for SB + vehicle).

In addition, microinjection of SB-258585 (4 μg) into the VLO, 10 min prior to WAY-208466 (8 μg) injection, completely antagonized the WAY-evoked inhibition of allodynia, as the PWT was reduced to the control level, as shown in Figures 4C,D. The time course curves of the PWT in vehicle + saline, vehicle + WAY, SB + WAY, and SB + saline treated groups were different between treatments [*F*_(__3_, _252__)_ = 38.08, *p* < 0.0001], across times [*F*_(__6_, _252__)_ = 6.564, *p* < 0.0001] and for treatment × time interaction [*F*_(__18_, _252__)_ = 2.911, *p* = 0.0001]. Detailed comparisons at individual time points between the various groups are shown in Figure 4C (1.987 ± 0.257, 2.163 ± 0.282, 2.096 ± 0.310, 2.161 ± 0.393, 2.291 ± 0.373, 2.453 ± 0.292, and 1.824 ± 0.291 for vehicle + saline group; 2.388 ± 0.226, 5.915 ± 0.779, 5.903 ± 0.677, 4.421 ± 0.627, 4.011 ± 0.436, 3.235 ± 0.356, and 3.231 ± 0.287 for vehicle + WAY group; 2.603 ± 0.172, 3.411 ± 0.372, 3.705 ± 0.495, 2.754 ± 0.222, 2.791 ± 0.195, 2.724 ± 0.209, and 2.985 ± 0.144 for SB + WAY group; 2.421 ± 0.413, 2.577 ± 0.356, 2.726 ± 0.342, 2.468 ± 0.315, 2.612 ± 0.295, 2.292 ± 0.341, and 2.739 ± 0.168 for SB + saline group). Further analyses indicated that the AUC in the SB + WAY group was significantly smaller than that in the vehicle + WAY group (*p* < 0.01), and no significant difference was found between SB + WAY and vehicle + saline groups, as shown in Figure 4D (130.758 ± 12.930 for vehicle + saline group; 262.997 ± 24.083 for vehicle + WAY group; 181.920 ± 13.509 for SB + WAY group; 174.850 ± 8.951 for SB + saline group).

Microinjection of SB-258585 (1, 2, and 4 μg) into the VLO had no effect on allodynia induced by SNI, as the PWT was maintained at the control level (*p* > 0.05, Figure 4E (2.068 ± 0.309, 2.302 ± 0.329, 2.401 ± 0.287, 2.497 ± 0.397, 2.554 ± 0.402, 2.752 ± 0.261, and 2.130 ± 0.255 for vehicle group; 2.708 ± 0.241, 2.823 ± 0.221, 2.865 ± 0.219, 2.896 ± 0.229, 2.703 ± 0.206, 2.849 ± 0.207, and 2.925 ± 0.242 for 1 μg SB group; 3.065 ± 0.144, 3.108 ± 0.200, 2.733 ± 0.158, 2.825 ± 0.257, 2.752 ± 0.217, 3.008 ± 0.154, and 3.023 ± 0.141 for 2 μg SB group; 2.421 ± 0.413, 2.577 ± 0.356, 2.726 ± 0.342, 2.468 ± 0.315, 2.612 ± 0.295, 2.292 ± 0.341, and 2.739 ± 0.168 for 4 μg SB group) and Figure 4F (130.758 ± 12.930 for vehicle group; 169.608 ± 12.167 for 1 μg SB group; 174.850 ± 8.951 for 2 μg SB group; 152.618 ± 17.212 for 4 μg SB group).

### Blocking Effect of AC Inhibitor SQ and PKA Inhibitor H89 on EMD-Induced Anti-allodynia

**FIGURE 5:**
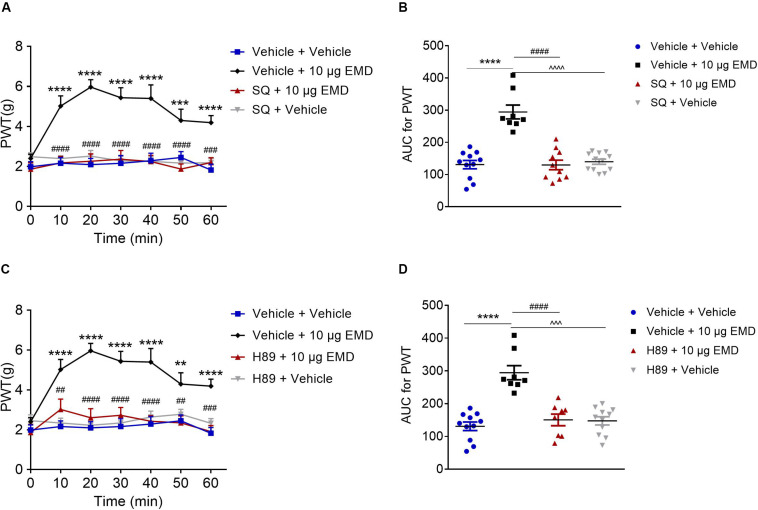
The blocking effects of the AC inhibitor SQ and PKA inhibitor H89 on EMD-induced anti-allodynia in the VLO. **(A,C)** Time course curves showing the blocking effects of SQ and H89 on EMD-induced inhibition of allodynia. **(B,D)** Scatter plots showing the AUC for the PWT for the blocking effect of SQ and H89 on EMD-induced inhibition of allodynia during the 60-min observation period. **p < 0.01, ***p < 0.001, and ****p < 0.0001, compared with vehicle group; ^##^*p* < 0.01, ^###^*p* < 0.001, and ^####^*p* < 0.0001 compared with 10 μg EMD. *n* = 8–12 rats/group.

Pretreatment of AC inhibitor SQ-22536 (2 nmol) into the VLO did not influence allodynia induced by SNI. However, when the same dose of SQ with EMD-386088 (10 μg) was applied to the VLO, SQ significantly blocked the EMD-evoked inhibition of allodynia (Figures 5A,B). As shown in Figure 5A, the time course curves of the PWT in vehicle + vehicle, vehicle + EMD, SQ + EMD, and SQ + vehicle treated groups were different between treatments [*F*_(__3_, _222__)_ = 27.08, *p* < 0.0001], across times [*F*_(__6_, _222__)_ = 7.434, *p* < 0.0001], and for treatment × time interaction [*F*_(__18_, _222__)_ = 4.462, *p* < 0.0001]. The detailed comparisons at individual time points are shown in Figure 5A (1.987 ± 0.257, 2.163 ± 0.282, 2.096 ± 0.310, 2.161 ± 0.393, 2.291 ± 0.373, 2.453 ± 0.292, and 1.824 ± 0.291 for vehicle + vehicle group; 2.401 ± 0.215, 5.024 ± 0.510, 5.969 ± 0.371, 5.440 ± 0.500, 5.401 ± 0.680, 4.299 ± 0.566, and 4.191 ± 0.351 for vehicle + EMD group; 1.865 ± 0.317, 2.180 ± 0.320, 2.261 ± 0.371, 2.368 ± 0.438, 2.244 ± 0.310, 1.870 ± 0.258, and 2.196 ± 0.243 for SQ + EMD group; 2.492 ± 0.195, 2.401 ± 0.143, 2.518 ± 0.283, 2.281 ± 0.217, 2.259 ± 0.224, 2.173 ± 0.133, and 2.187 ± 0.205 for SQ + vehicle group). The AUC of the PWT over the 60-min observation period in the SQ + EMD group was significantly smaller than that in the vehicle + EMD group (*p* < 0.0001), while the PWT was reduced to the control level [*p* > 0.05, Figure 5B (130.758 ± 12.930 for vehicle + vehicle group; 294.375 ± 21.543 for vehicle + EMD group; 129.630 ± 14.896 for SQ + EMD group; 139.803 ± 7.940 for SQ + vehicle group)].

Furthermore, microinjection of the PKA inhibitor H89 (10 nmol) into the VLO did not influence allodynia induced by SNI. However, when the same dose of H89 with EMD-386088 (10 μg) was applied into the VLO, the H89 significantly blocked the EMD-evoked inhibition of allodynia (Figures 5C,D). As shown in Figure 5C, time course curves of PWT in vehicle + vehicle, vehicle + EMD, H89 + EMD, and H89 + vehicle treated groups were different between treatments [*F*_(__3_, _238__)_ = 69.75, *p* < 0.0001], across times [*F*_(__6_, _238__)_ = 4.981, *p* < 0.0001], and for treatment × time interaction [*F*_(__18_, _238__)_ = 2.533, *p* = 0.0008]. The detailed comparisons at individual time points are shown in Figure 5C (1.987 ± 0.257, 2.163 ± 0.282, 2.096 ± 0.310, 2.161 ± 0.393, 2.291 ± 0.373, 2.453 ± 0.292, and 1.824 ± 0.291 for vehicle + vehicle group; 2.401 ± 0.215, 5.024 ± 0.510, 5.969 ± 0.371, 5.440 ± 0.500, 5.401 ± 0.680, 4.299 ± 0.566, and 4.191 ± 0.351 for vehicle + EMD group; 1.863 ± 0.408, 3.024 ± 0.520, 2.604 ± 0.458, 2.731 ± 0.395, 2.431 ± 0.280, 2.346 ± 0.348, and 1.910 ± 0.306 for H89 + EMD group; 2.457 ± 0.292, 2.343 ± 0.237, 2.226 ± 0.216, 2.343 ± 0.316, 2.642 ± 0.292, 2.778 ± 0.248, and 2.326 ± 0.235 for H89 + vehicle group). The AUC of the PWT over the 60-min observation period in the H89 + EMD group was significantly smaller than that in the vehicle + EMD (10 μg) group (*p* < 0.0001), but no significant difference was found between H89 + EMD, and vehicle + EMD groups (*p* > 0.05), as shown in Figure 5D (130.758 ± 12.930 for vehicle + vehicle group; 294.375 ± 21.543 for vehicle + EMD group; 150.334 ± 17.745 for H89 + EMD group; 147.323 ± 12.603 for H89 + vehicle group).

### Blocking Effect of a Glutamate Receptor Antagonist on EMD-Induced Anti-allodynia

**FIGURE 6:**
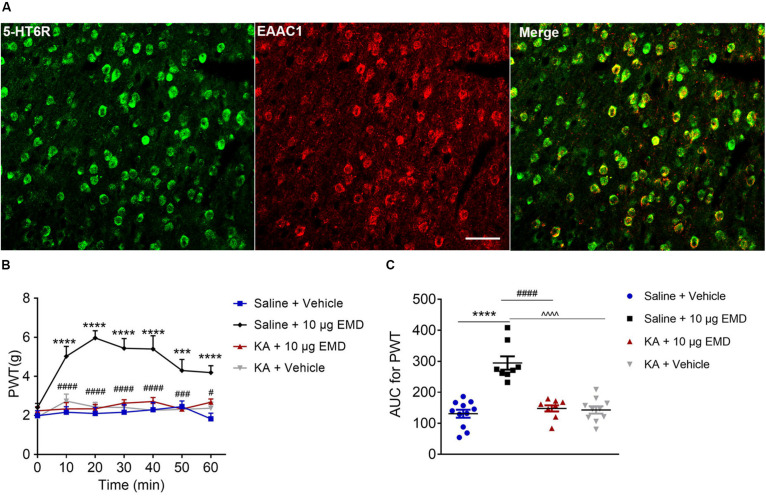
The blocking effects of the non-selective glutamate receptor antagonist KA on EMD-induced anti-allodynia in the VLO. **(A)** The colocalization of 5-HT_6_ receptors and EAAC1 in the VLO. Scale bar, 50 μm; *n* = 4 rats/group. **(B)** Time course curves showing the antagonizing effect of KA on EMD-induced inhibition of allodynia. **(C)** Scatter plots showing the antagonizing effect of KA on EMD-induced inhibition of allodynia during the 60-min observation period. ***p < 0.001 and ****p < 0.0001, compared with vehicle group; ^#^*p* < 0.05, ^###^*p* < 0.001, and ^####^*p* < 0.0001 compared with 10 μg EMD. *n* = 8–11 rats/group.

Thus far, we have demonstrated the intracellular molecular pathway underlying 5-HT_6_ receptor agonists’ anti-allodynic effect. Considering that the VLO is an indispensable part of the analgesic system, with activation of the VLO attenuating allodynia, and that activation of 5-HT_6_ receptors inhibited allodynia, we speculated that 5-HT_6_ receptors might interact with excitatory neurons. To test this hypothesis, we first conducted immunostaining of 5-HT_6_ receptors and glutamate transporter (EAAC1), as previous immunoreactivity of EAAC1 corresponds to what has been described for glutamatergic neuronal systems (Wernig et al., 2004; Liu et al., 2019). Indeed, we found a fair amount of colocalization of 5-HT_6_ receptors and EAAC1, as shown in Figure 6A. These results implied that 5-HT_6_ receptor agonists might exhibit anti-allodynic effect potentially (but not exclusively, Supplementary Figure S4) via interaction with the glutamatergic system.

To confirm the hypothesis that 5-HT_6_ receptors could interact with glutamatergic system, we next microinjected glutamate receptor antagonist KA into the contralateral VLO. As shown in Figures 6B,C, KA (0.76 μg) significantly blocked the EMD-evoked inhibition of allodynia. As shown in Figure 6B, the time course curves of PWT in saline + vehicle, saline + EMD, KA + EMD, and KA + vehicle treated groups were different between treatments [*F*_(__3_, _204__)_ = 25.81, *p* < 0.0001], across times [*F*_(__6_, _204__)_ = 7.958, *p* < 0.0001], and treatment × time interaction [*F*_(__18_, _204__)_ = 4.088, *p* < 0.0001]. Detailed comparisons at individual time points are shown in Figure 6B (1.987 ± 0.257, 2.163 ± 0.282, 2.096 ± 0.310, 2.161 ± 0.393, 2.291 ± 0.373, 2.453 ± 0.292, and 1.824 ± 0.291 for saline + vehicle group; 2.401 ± 0.215, 5.024 ± 0.510, 5.969 ± 0.371, 5.440 ± 0.500, 5.401 ± 0.680, 4.299 ± 0.566, and 4.191 ± 0.351 for saline + EMD group; 2.236 ± 0.212, 2.332 ± 0.323, 2.339 ± 0.231, 2.624 ± 0.178, 2.712 ± 0.203, 2.323 ± 0.210, and 2.672 ± 0.170 for KA + EMD group; 1.921 ± 0.205, 2.731 ± 0.362, 2.431 ± 0.241, 2.409 ± 0.261, 2.265 ± 0.403, 2.304 ± 0.266, and 2.369 ± 0.313 for KA + vehicle group). The AUC of the PWT over the 60-min observation period in the KA + EMD group was significantly smaller than that in the Saline + EMD group (*p* < 0.0001), where the PWT was reduced to the control level [*p* > 0.05, Figure 6C (130.758 ± 12.930 for saline + vehicle group; 294.375 ± 21.543 for saline + EMD group; 147.952 ± 9.914 for KA + EMD group; 142.927 ± 11.906 for KA + vehicle group)]. These results indicated that 5-HT_6_ receptor agonists’ anti-allodynic effect was dependent on glutamate release.

## Discussion

Mechanical allodynia, which is characterized by a painful sensation induced by innocuous stimuli such as light touch (Jensen and Finnerup, 2014; Lolignier et al., 2015), is thought to be caused by disruption in pain-related regions. Identification and reversal of any associated pathologic neuroadaptations are therefore beneficial for clinical treatment. Mounting evidence has established that the VLO, as a cerebral higher center of an endogenous analgesic system comprising the spinal cord–Sm–VLO–PAG–spinal cord (Tang et al., 2009), is involved in the modulation of nociception. Specifically, the Sm located in the medial thalamus receives major projections from the trigeminal subnucleus caudalis and the spinal dorsal, and projects primarily to the VLO. The VLO contains neurons that project to the PAG. Therefore, the Sm, VLO, and PAG constitute a pain modulatory pathway, peripheral or local activation of which results in activation of the PAG–brainstem descending inhibitory system and depression of the nociceptive inputs in the spinal cord. Diverse types of transmitters and their corresponding receptors, such as opiates and their receptors, 5-HT and its receptors, dopamine and its receptors, GABA and its receptors, glutamate and its receptors, noradrenaline and its receptors, have been delineated within the VLO (Qu et al., 2006; Huo et al., 2009; Wei et al., 2016). Depending on the location of the specific receptors at different types of neurons, they may interact with each other and play similar or opposite effects. For example, activation of μ-opioid receptor, 5-HT_1__A_ receptor and D_2_-like dopamine receptor via the increase of endogenous opioid peptide, 5-HT and dopamine release presynaptically inhibited the tonic inhibitory action of GABAergic terminals on the neurons projecting to VLO, such a disinhibitory effect leads to activation of the Sm–VLO–PAG brainstem descending inhibitory system and produces antinociception. The present study aimed to dissect the individual effect of 5-HT_6_ receptors and their interactive substrate within the VLO. Our previous study showed that 5-HT microinjection into the VLO depressed SNI-induced allodynia and that this anti-allodynic effect was significantly antagonized by the selective 5-HT_6_ receptor antagonist SB-258585 (Xu et al., 2013). Although this emerging evidence suggested that 5-HT_6_ receptors might be involved in VLO-modulated neuropathic pain, how 5-HT_6_ receptor dysfunction leads to this pain remains poorly understood. To our knowledge, the present study unveils the role of 5-HT_6_ receptors in the VLO in modulating neuropathic pain for the first time.

It has been established that a majority of the nociceptive inputs from the spinal cord are transmitted to the thalamus, and collateral projections also target mesencephalic nuclei, including the DRt, the RVM, and the midbrain PAG (Ossipov et al., 2010). Among these critical pain modulation sites, the VLO is a pivotal component (Tang et al., 2009). However, much less is known regarding this modulation with respect to the molecular manifestations of neuropathic pain. To address this question, we first performed Western blotting to compare the expression level of 5-HT_6_ receptors in the VLO. As our results showed, 5-HT_6_ receptors were decreased specifically in the VLO contralateral to the nerve injury. These findings were consistent with the nociceptive modulation loop, which demonstrated that most of the nociceptive signals transmitted to the contralateral VLO (Tang et al., 2009). Importantly, these findings also revealed that 5-HT_6_ receptor hypofunction might arise from SNI-induced mechanical allodynia. As a consequence, activation of 5-HT_6_ receptors by both agonists consistently attenuated allodynia. Interestingly and counterintuitively, although the 5-HT_6_ receptors markedly decreased after SNI, the exogenous ligand could still produce pronounced anti-nociception via activating the existing receptors. The present study showed that microinjection of 5-HT_6_ receptor agonist affected allodynia only in SNI induced rats, not for sham animals. We speculated that in sham or naïve rats, there is always a balanced steady state, so that agonist or antagonist will not take effect. However, SNI dramatically decreased the protein expression of 5-HT_6_ receptors, and as a consequence, the homeostatic state was disrupted. The existing 5-HT_6_ receptors in SNI rats are more sensitive and vulnerable to exogenous agonist treatment, so that agonist could induce anti-allodynia. The VLO received projections from spinal cord as well as contained output neurons that projected to the PAG (Tang et al., 2009; Qu et al., 2015). Therefore, it is reasonable to propose that the anti-allodynic effects elicited by 5-HT_6_ receptor agonists are due to activation of the VLO neurons that project to the PAG, which further leads to activation of the PAG–brainstem descending inhibitory system and depresses the nociceptive transmission at the spinal cord level. Notably, we observed that there was no clear effect on allodynia when SB-258585 was administered alone into the VLO, which was consistent with the results of a previous study (Xu et al., 2013). It is not difficult to understand this phenomenon, as the 5-HT_6_ receptors were dramatically decreased after the development of allodynia and the PWT already reached the lower limit, i.e., 2.

Recently, 5-HT_6_ receptors have prompted great interest due to their particularly promising potential for the treatment of various neuropsychiatric diseases such as Alzheimer’s disease and schizophrenia, consistent with their highest expression in regions involved in cognitive functions (de Bruin and Kruse, 2015; de Jong and Mork, 2017). The diversity of functions modulated by 5-HT_6_ receptors reflects not only their capabilities to elicit different signal transduction mechanisms, but also their propensity to interact with both canonical (G-dependent) and non-conventional signaling (Kim et al., 2019). As for the possible intracellular mechanism of 5-HT_6_ receptor agonist in the VLO-mediated nociceptive modulation, the AC inhibitor SQ or PKA inhibitor H89 was microinjected into the VLO prior to EMD injection in the present study. We observed that both inhibitors almost completely blocked the effects of EMD-induced anti-allodynia, thus suggesting that the AC-PKA pathway in the VLO might participate in 5-HT_6_ receptor modulation of neuropathic pain.

**FIGURE 7:**
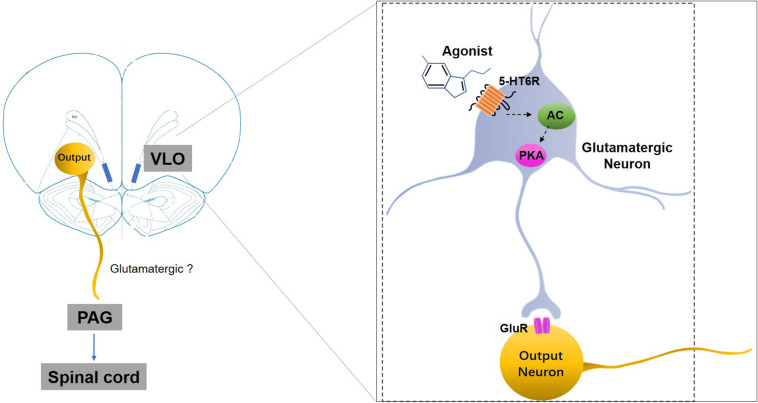
A schematic representation of the mechanisms underlying 5-HT_6_ receptors’ regulation of SNI-induced mechanical allodynia. SNI-induced allodynia downregulates 5-HT_6_ receptors specifically in the contralateral VLO. The downregulation of 5-HT_6_ receptors, some of which are postsynaptically present in the glutamatergic neurons in the VLO, might subsequently decrease glutamate release via an AC/PKA cascade, thus resulting in allodynia. Therefore, selective 5-HT_6_ receptor agonists may ameliorate some of these mal-adaptations by activation of 5-HT_6_ receptors. GluR, glutamate receptors; 5-HT6R, 5-HT_6_ receptors.

We also observed that the anti-allodynic effects induced by 5-HT_6_ receptor activation could be blocked by microinjection of the glutamate receptor antagonist (KA) into the VLO. It is well documented that the 5-HT_6_ receptors are a type of excitatory Gs protein coupled receptor, and that activation of this receptor type increases neuronal activity (Ruat et al., 1993). In addition, microinjection of the 5-HT_6_ receptor agonist WAY-208466 into the rat medial prefrontal cortex, specifically the prelimbic cortex, increased the firing rate of the glutamatergic neurons in both normal controls and Parkinson model rats, while microinjection of the 5-HT_6_ receptor antagonist SB-258585 decreased the firing rate of the neurons (Zhang et al., 2016). Thus, we speculated that the excitatory effect on the VLO neurons induced by activation of 5-HT_6_ receptors was due to the 5-HT_6_ receptors’ interaction with glutamatergic systems. Activation of 5-HT_6_ receptors (most postsynaptically), some of which are located on glutamatergic neurons, facilitated glutamate release, thereby indirectly exciting the VLO output neurons projecting to the PAG to induce anti-nociception (Figure 7).

Notably, several studies have indicated that local peripheral and central 5-HT_6_ receptors play a pronociceptive role in formalin-induced nociceptive behavior after subcutaneous, intraperitoneal or intrathecal administration (Castaneda-Corral et al., 2009; Godinez-Chaparro et al., 2011; Zhang et al., 2019). Several hypotheses for this discrepancy can be proposed. First and foremost, 5-HT_6_ receptor-related compounds may act on different receptor populations located in different regions of the rat (Dawson et al., 2001; King et al., 2008; Upton et al., 2008; Tassone et al., 2011; Ramírez, 2013; Zhang et al., 2016). The stimulation of receptors located on glutamatergic and cholinergic neurons by the agonist, leading to an increase in both glutamate and acetylcholine liberation, versus the action of an agonist on the receptor located on GABAergic interneurons, would produce opposite behavioral responses. The present study, for example, as mentioned above, demonstrated that the anti-allodynic effect was an explicit consequence of a strong excitatory (glutamatergic) drive to the PAG. However, we cannot exclude the possibility that 5-HT_6_ receptor agonists activated both glutamatergic neurons and GABAergic interneurons, while the E/I balance still favored the excitatory effect. Second, different types of nociceptive stimuli as well as the experimental approach used may account for the discrepancy. However, it is more likely that the different nociceptive stimuli and models of pain may be responsible, as preliminary formalin studies measured flinching behavior as inflammatory nociception, characterized by spontaneous pain, while the SNI model (the present study) assessed mechanical allodynia-like behavior of persistent neuropathic pain (Decosterd and Woolf, 2000; Bourquin et al., 2006). As 5-HT_6_ receptors are largely but not exclusively colocalized with glutamatergic neurons in the VLO and as different ligands may bias specific cell types at specific pathologic settings, future studies could focus on elucidating the cell-type (e.g., excitatory vs inhibitory) contribution to different types of pain.

In summary, the present study reported that activation of 5-HT_6_ receptors in the VLO regulated allodynia in a rodent model of neuropathic pain via glutamatergic neurotransmission, as well clarifying the AC/PKA signal pathway. Based on these findings, the present study uncovered a potential therapeutic role of 5-HT_6_ receptor agonists in the context of neuropathic pain.

### Major Unresolved Questions

Although we have provided strong evidence for the involvement of 5-HT_6_ receptor signaling in neuropathic pain, current methodological limitations made it difficult to parse out the contribution of certain neuronal subpopulations in refining cortical sensory processing. Besides, in the present study the 5-HT_6_ agonist was microinjected into the VLO rather than systemically administered. From clinic perspective, it should be acknowledged that systematic administration might risk potential off-target and contradictory or even unwanted effects, which should be addressed in the future therapeutic development.

## Data Availability Statement

The raw data supporting the conclusions of this article will be made available by the authors, without undue reservation.

## Ethics Statement

The animal study was reviewed and approved by Institutional Animal Care Committee of Xi’an Jiaotong University.

## Author Contributions

YZ, FH, and CY designed the research. YZ performed the experiments. XY, YWu, JL, and YWa provided technical support and performed the surgery. YZ and JY analyzed the data. YZ and CY wrote the manuscript and FH edited it. All authors approved the manuscript for publication.

## Conflict of Interest

The authors declare that the research was conducted in the absence of any commercial or financial relationships that could be construed as a potential conflict of interest.
