# Supplementary material for: The 5-HT6 Receptors in the Ventrolateral Orbital Cortex Attenuate Allodynia in a Rodent Model of Neuropathic Pain
Source: Front Neurosci. 2020 Aug 18;14:884. doi: 10.3389/fnins.2020.00884 (PMC7461796; doi:10.3389/fnins.2020.00884)

Supplementary Material

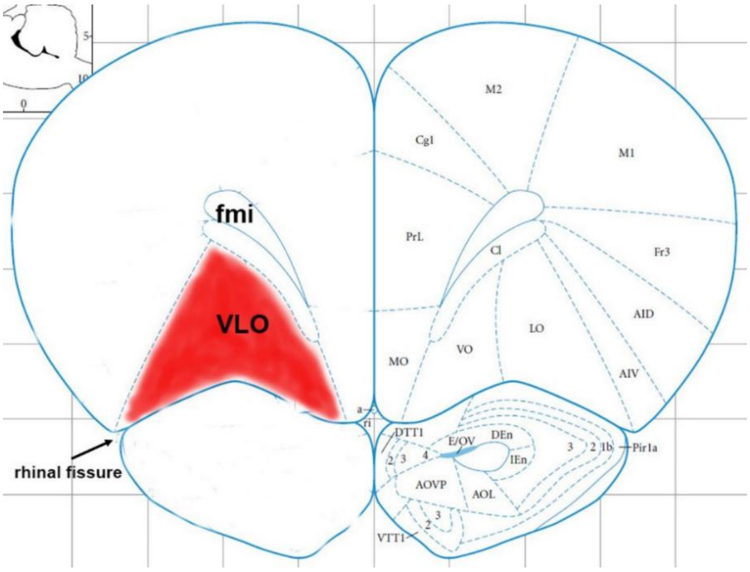

Fig. S1. A schematic representation of the size of VLO (bregma +4.2). The VLO is surrounded by rhinal fissure and fmi.

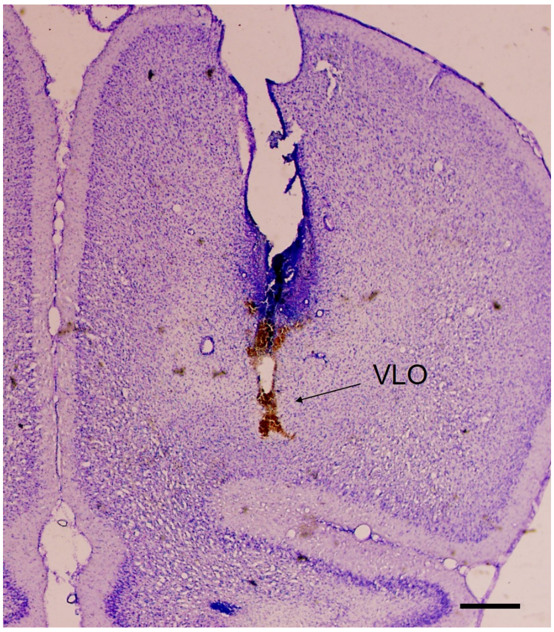

Fig. S2. Photomicrograph examples of an injection site in the VLO. Arrows indicate the location of injection site within VLO. Abbreviations: VLO, ventrolateral orbital cortex. Scale bar = 500 µm.

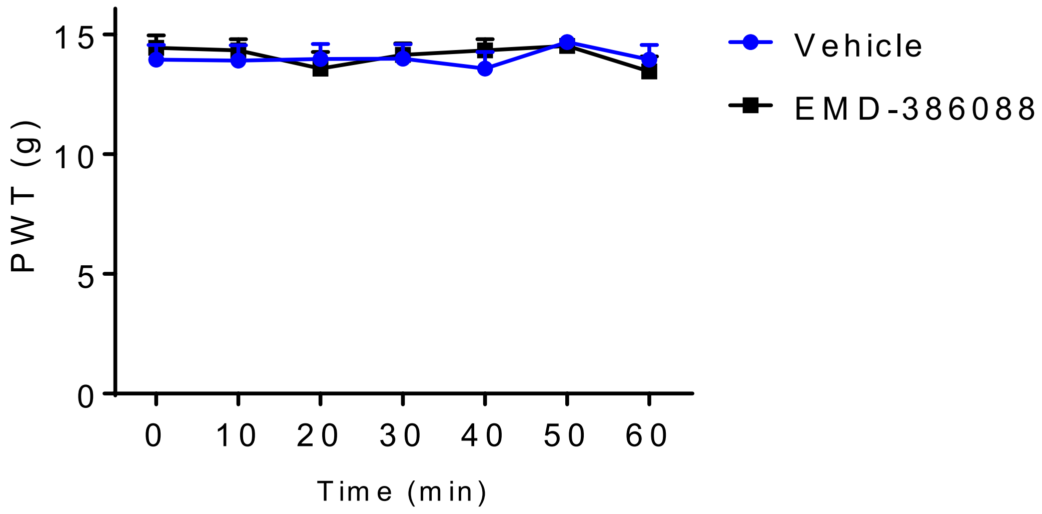

Fig. S3. Time-course curves showing no effect of 5-HT_6 receptor agonist EMD-386088 on PWT in sham rats. n = 6 rats / group.

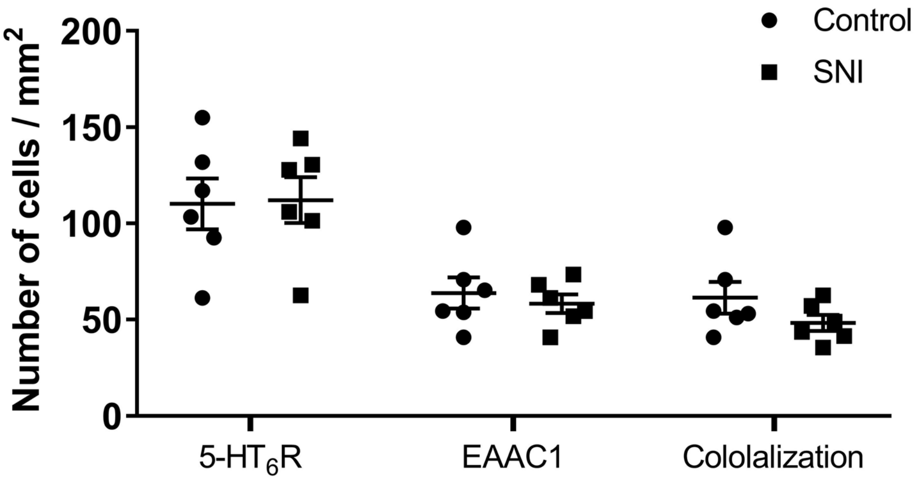

Fig. S4. Scatter plots showing the number of 5-HT_6R-positive, EAAC1-positive and colocalization neurons per mm^2 in the VLO. n = 3 rats / group.

Results

1. Two-way ANOVA analysis revealed no significant effect between time ($F_{(6, 70)}$ = 0.5794, p = 0.7455), treatment ($F_{(1, 70)}$ = 0.1398, p = 0.7096) or interaction ($F_{(6, 70)}$ = 0.3705, p = 0.8953, **Fig. S3**).
2. Two-way ANOVA analysis revealed significant effect between rows ($F_{(2, 15)}$ = 21.03, p < 0.0001), but not treatment or interaction ($F_{(1, 15)}$ = 0.6432, p = 0.4351, SNI treatment factor; $F_{(2, 15)}$ = 0.3895, p = 0.6840, interaction). Student t-test showed that SNI treatment had no effect on the positive neurons (p > 0.05, 5-HT_6R (110.204 ± 13.284 for Control and 112.132 ± 11.869 for SNI); p > 0.05, EAAC1 (63.832 ± 8.031 for Control and 58.277 ± 4.822 for SNI); p > 0.05, colocalization (61.338 ± 8.314 for Control and 48.186 ± 4.158 for SNI), **Fig. S4**).

Discussion Notes

It is not difficult to understand that the positive cell numbers had no change after SNI treatment. In other words, pathological state (neuropathic pain) decreased 5-HT_6 receptor expression volume, but not the cell identity.

Full, untruncated images of Western blotting

Fig. 2

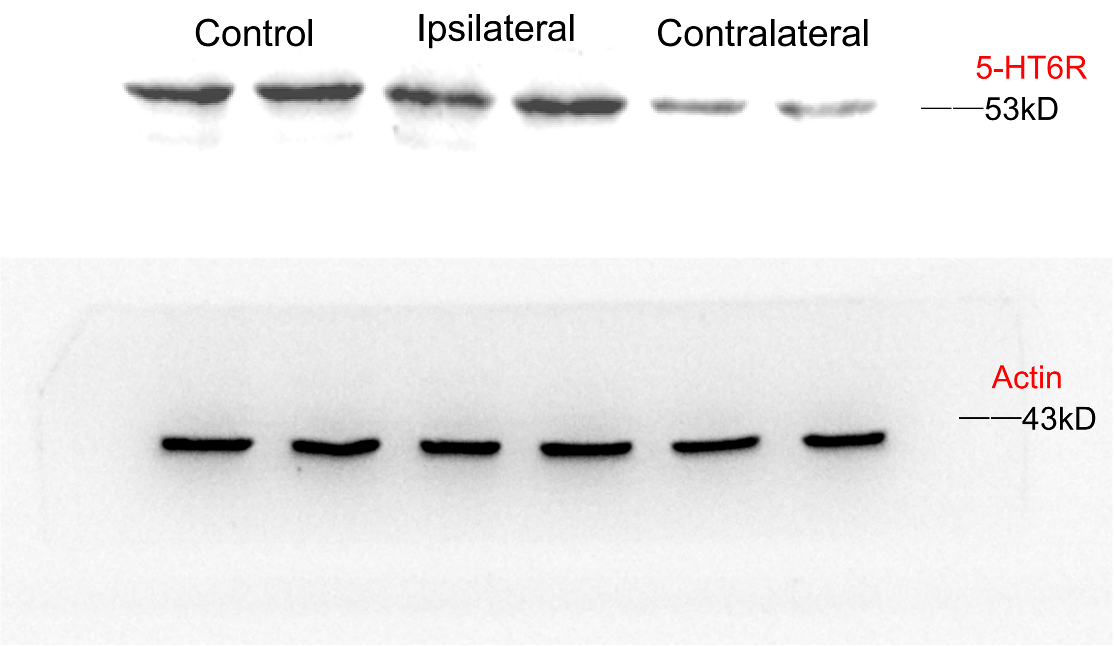

Supplement: Supplementary file 1 [file Data_Sheet_1.pdf]
